# The Effect of Foot Reflexology on Chemotherapy-Induced Nausea and Vomiting in Patients With Digestive or Lung Cancer: Randomized Controlled Trial

**DOI:** 10.2196/25648

**Published:** 2021-11-05

**Authors:** Audrey Murat-Ringot, Pierre Jean Souquet, Fabien Subtil, Florent Boutitie, Marie Preau, Vincent Piriou

**Affiliations:** 1 Hôpital Lyon Sud Hospices Civils de Lyon Pierre-Bénite France; 2 INSERM U1290 Research on Healthcare Performance Claude Bernard University I Lyon France; 3 Groupe de Recherche en Psychologie Sociale EA 4163 Institut de Psychologie Université Lyon 2 Bron France; 4 Pôle Santé Publique - Service de Biostatistiques Hospices Civils de Lyon Lyon France; 5 Biometrics and Evolutionary Biology UMR 5558 Claude Bernard University I Centre national de la recherche scientifique Villeurbanne France

**Keywords:** cancer, randomized controlled trial, foot reflexology, nausea and vomiting, chemotherapy, complementary and alternative medicine

## Abstract

**Background:**

Cancer is a chronic disease with an incidence of 24.5 million and 9.6 million deaths worldwide in 2017. Lung and colorectal cancer are the most common cancers for both sexes and, according to national and international recommendations, platinum-based chemotherapy is the reference adjuvant treatment. This chemotherapy can be moderately to highly emetogenic. Despite antiemetic therapy, chemotherapy-induced nausea and vomiting (CINV) may persist. Moreover, cancer patients are increasingly interested in alternative and complementary medicines and have expressed the desire that nonpharmacological treatments be used in hospitals. Among alternative and complementary medicines, foot reflexology significantly decreases the severity of CINV in patients with breast cancer.

**Objective:**

The primary aim of this study was to assess the benefits of foot reflexology as a complement therapy to conventional treatments regarding the severity of acute CINV in patients with digestive or lung cancer. The secondary objectives assessed were the frequency and severity of delayed CINV, quality of life, anxiety, and self-esteem.

**Methods:**

This study was conducted between April 2018 and April 2020 in the Hospices Civils de Lyon, France. This was an open-label randomized controlled trial. Participants were randomized into two groups: the intervention group (ie, conventional care with foot reflexology; n=40) and the control group (ie, conventional care without foot reflexology; n=40). Foot reflexology sessions (30 minutes each) were performed on outpatients or inpatients. Eligible participants were patients with lung or digestive cancer with an indication for platinum-based chemotherapy.

**Results:**

The severity of acute nausea and vomiting was assessed with a visual analog scale during the second cycle of chemotherapy. A significant increase of at least 2 points was observed for the control group (7/34, 21%; *P*=.001). Across all cycles, the foot reflexology group showed a trend toward less frequent delayed nausea (*P*=.28), a significantly less frequent consumption of antiemetic drugs (*P*=.04), and no significant difference for vomiting (*P*=.99); there was a trend toward a perception of stronger severity for delayed nausea in the control group (*P*=.39). Regarding quality of life and anxiety, there was no significant difference between the intervention group and the control group (*P*=.32 and *P*=.53, respectively).

**Conclusions:**

This study’s results indicate that foot reflexology provides significantly better management of acute nausea severity and decreased consumption of antiemetic drugs in patients with lung or digestive cancer. In order to fulfill patients’ desires to use nonpharmacological treatments and complementary and alternative medicines in hospitals, foot reflexology could be provided as a complementary intervention to conventional antiemetic drugs. Foot reflexology did not result in adverse effects. To assess the benefits of foot reflexology in routine practice, a larger study with several health care centers would be needed with a cluster randomized controlled trial.

**Trial Registration:**

ClinicalTrials.gov NCT03508180; https://clinicaltrials.gov/ct2/show/NCT03508180

**International Registered Report Identifier (IRRID):**

RR2-10.2196/17232

## Introduction

According to estimates made by the Global Cancer Observatory, lung cancer was the most common cancer for both sexes in 2018 (11.6% of the total number of cancers), followed closely by breast cancer (11.6%), prostate cancer (7.1%), and colorectal cancer (6.1%); the leading cause of cancer death was lung cancer (18.4% of total cancer deaths), followed by colorectal cancer (9.2%), stomach cancer (8.2%), and liver cancer (8.2%) [1]. Platinum-based chemotherapy is the adjuvant treatment for lung and digestive cancers according to national and international recommendations [2-7]. Cisplatin is a highly emetogenic chemotherapy (ie, the occurrence of chemotherapy-induced nausea and vomiting [CINV] >90%), while carboplatin and oxaliplatin are moderately emetogenic chemotherapies (ie, incidence of CINV ranges from 30% to 90%) [8]. CINV can either be acute (ie, occurring within 24 hours of receiving chemotherapy) or delayed (ie, occurring between 2 and 5 days following treatment) [8]. It is the side effect most feared by patients, decreasing their overall quality of life [9-12], and may lead to metabolic complications [13]. In addition, CINV can lead to dose reduction, postponement of treatment, and even discontinuation [14], which can decrease the effectiveness of treatment [15]. To prevent and control both acute and delayed CINV, antiemetic drugs are prescribed; the main ones used are 5-hydroxytrytamine 3 receptor antagonists, dexamethasone, and neurokinin-1 receptor antagonists [8,13]. While vomiting is well controlled, nausea remains a significant problem in practice [16]. In addition to the emetogenicity of the chemotherapy, various parameters may also lead to CINV, including risk factors (ie, age, sex, alcohol use, history of motion sickness, and history of pregnancy-related vomiting) [10], antiemetic treatment adherence [17], and the gap in perception of CINV between health professionals and patients [18,19].

To treat their cancer and the side effects of treatment, as well as to improve quality of life, patients with cancer are increasingly using complementary and alternative medicines (CAMs) [20,21]. According to a European survey reported by Molassiotis et al, 35.9% of patients with cancer use CAMs [21]. For various reasons, some patients do not inform the caregivers that they use CAMs [22,23]; however, certain CAMs may potentially interact with conventional cancer treatments [24,25]. According to the citizen science study reported by Tran et al, in France, patients with chronic disease, including cancer, have clearly expressed a desire for nonpharmacological treatments and CAMs to be used in hospitals to improve their care [26]. In parallel, oncologists lack information about the safety and efficacy of CAMs to inform their patients [27-29] and they request more rigorous evaluation [28,29]. Among the most frequently provided CAMs in private and public oncology centers in European countries [30], foot reflexology seems very interesting. Foot reflexology involves applying pressure to specific areas of the feet, which helps the body restore homeostasis. The premise is that reflex zones in the feet correspond to organs, glands, and systems of the body [31]. Foot reflexology used concomitantly with conventional treatment seems to decrease some side effects induced by chemotherapy; more specifically, this combination improves quality of life [32,33], significantly decreases pain intensity and anxiety in patients with metastatic cancer [34], and significantly improves the perceived pain and anxiety in postoperative patients with gastric cancer and hepatocellular cancer [35]. Moreover, a significant decrease in CINV has been observed in patients with breast cancer receiving chemotherapy and foot reflexology [36,37]. But these studies were conducted among women only, whereas female sex is a risk factor for CINV [38,39]. In addition, the design of these studies did not provide a high level of evidence, a point underlined by systematic reviews that conclude that there is a necessity to confirm these results by randomized controlled trials (RCTs) [40,41].

Our primary hypothesis is that foot reflexology performed in association with conventional care will improve the management of acute nausea. Thus, the aim of this RCT is to determine whether foot reflexology provides better control of CINV in patients with lung or digestive cancer who are receiving platinum-based chemotherapy.

## Methods

### Trial Design

The REFYO-R (Reflexology/Yoga–Reflexology trial) study is an open-label RCT, the protocol of which has been published elsewhere [42]. Briefly, the patients were randomized to either conventional care with foot reflexology or conventional care without foot reflexology at a ratio of 1:1. This report followed the CONSORT Statement for Randomized Trials of Nonpharmacologic Treatments [43]. This study was approved by the regional ethics committee (Comité de Protection des Personnes Île de France X) on April 3, 2018 (ID No. RCB 2018-A00571-54). Regarding clinical research supported by the Hospices Civils de Lyon, processing of personal data complied with the methodological recommendations of the MR001 reference established by the French Data Protection Authority, Commission Nationale de l’Informatique et des Libertés (No. 18-071). Enrollment started in June 2018. This study was registered with ClinicalTrials.gov (NCT03508180) on June 28, 2018.

### Participants

Participants were selected according to the following criteria:

1. Aged ≥18 years.
2. Had lung cancer (ie, non–small cell lung carcinomas, small cell lung cancer, squamous cell carcinoma, or mesothelioma lung cancer) or digestive cancer (ie, colorectal cancer, pancreatic cancer, or liver cancer) at stages IV, IIIB, IIIA, or II.
3. Patients on platinum-based chemotherapy with or without concomitant radiation therapy.
4. Had World Health Organization performance status of ≤2.
5. Patients affiliated with the national social security system or equivalent.
6. Patients able to complete the questionnaires (ie, comprehension of oral and written French language).
7. Gave written informed consent.

The exclusion criteria were (1) phlebitis, (2) vena cava syndrome, (2) weight loss of >5% in the 3 months before the inclusion date, (3) uncontrolled pain, (4) patients receiving morphine or morphine derivatives, (5) brain metastases, (6) patients receiving foot reflexology outside the study, and (7) patients under guardianship or curatorship, or having been deprived of his or her rights. Patients gave written informed consent before inclusion and randomization. Patients in the control group received two sessions of foot reflexology after completion of the study.

### Settings

The study was conducted between April 25, 2018, and April 8, 2020, at the university hospitals of Lyon (Hospices Civils de Lyon, France).

### Intervention

**Figure 1:**
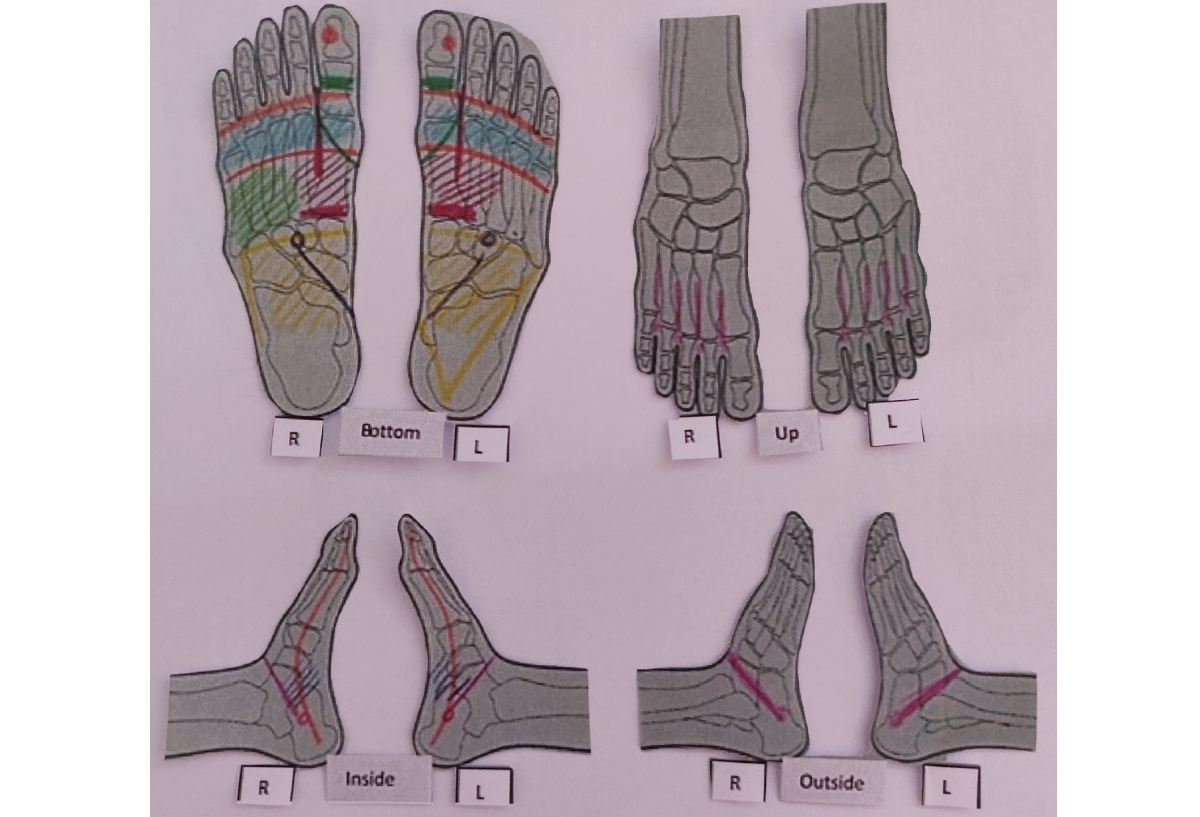
Reflex zones stimulated. L: left; R: right. (developed by C Rentler).

The patients randomized to the intervention group (n=40) received four sessions of foot reflexology (30 minutes each) during chemotherapy infusion every 2 or 3 weeks, according to the chemotherapy protocol. Three qualified reflexologists administered the sessions. The three reflexologists had same skills training approved by the French Federation of Reflexologists. The reflexology chart used in this clinical study is based on the one proposed by Eunice Ingham [31]. The intervention was standardized (Figure 1): to calm nausea and vomiting, the upper and lower digestive reflex points, as well as the metabolism of the smooth muscle reflex points (ie, lymphatic system, kidneys and bladder, lungs, thyroid, and parathyroid), were stimulated. To provide deep relaxation to target anxiety, the diencephalon reflex points, scapular belt reflex points, reflex points of the diaphragm, and reflex points of the spine were stimulated. After each stimulation of the reflex points, relaxation movements were performed [31].

**Figure 2:**
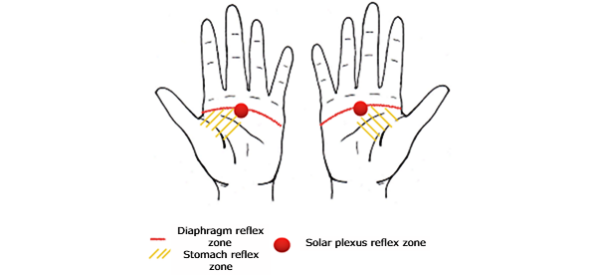
Self-massage diagram (developed by C Rentler).

During the first reflexology session, the reflexologist trained the patients in the foot reflexology group regarding the appropriate zones on the hands to relieve nausea. The reflexologist delivered to the patient a figure illustrating the palmar massage points (Figure 2).

All patients received standard antiemetic drugs (eg, 5-hydroxytryptamine 3 receptor antagonists, dexamethasone, and/or neurokinin-1 receptor antagonists) in accordance with guidelines [8,13].

### Adverse Events

All adverse events were collected during this study and the causality with foot reflexology was assessed by the oncologist.

### Outcome Measures

#### Primary Outcome

The primary outcome was the relative change in the severity of acute CINV, as assessed by a visual analog scale (VAS) during the second cycle of chemotherapy. The patient was asked to mark their current nausea level on the horizontal line, ranging from a happy face (minimum: no nausea = 0 mm) on the left to a very sick green face (maximum: paroxysm of nausea or vomiting = 100 mm) on the right. Unlike vomiting, which is measurable by the number of episodes per day, nausea is a subjective experience, the severity of which can be assessed using a VAS [44]. For those in the intervention group, this was measured before and after the foot reflexology session; for those in the control group, this was measured when the patient arrived at the outpatient or inpatient appointment and before leaving hospital.

#### Secondary Outcomes

The benefits of foot reflexology on delayed CINV were assessed using a diary completed every day by patients between the first and fourth cycle of chemotherapy. Every day, the patient assessed the frequency of nausea and vomiting, recording each emetic and nausea episode, and assessed the intensity of the worst nausea and vomiting episodes using a 6-point Likert scale with the following possible responses: 1 (“very low”), 2 (“low”), 3 (“moderate”), 4 (“severe”), 5 (“very severe”), and 6 (“unbearable”). Patients also recorded all rescue antiemetic medications, which were taken in addition to what was prescribed at baseline to prevent nausea and vomiting.

At baseline and at the end of the study period, the quality of life, anxiety, and self-esteem of participants were assessed. The score from the EORTC QLQ-C30 (European Organization for Research and Treatment of Cancer Quality of Life Questionnaire–Core 30) [45] was used to assess health-related quality of life. This questionnaire includes five functional scales (ie, physical, daily activity, emotional, cognitive, and social), three symptomatic scales (ie, fatigue, nausea and vomiting, and pain), six unique items relating to certain symptoms or problems (ie, dyspnea, insomnia, loss of appetite, constipation, diarrhea, and financial impact), and two global scales of health status and quality of life.

The Hospital Anxiety and Depression Scale (HADS) score [46] was used to assess anxiety; this scale has been validated in French [47,48] and consists of 14 items, including seven items each for the anxiety subscale (HADS-A) and the depression subscale (HADS-D). As a self-rating scale, its scoring system ranged from the absence of symptoms (score of 0) to the maximal presentation of symptoms (score of 3).

To assess self-esteem, the Body Image Questionnaire (BIQ) [49-51] was used at the end of the study and was compared to the level of self-esteem assessed with the Rosenberg Self-Esteem Scale (RSES) administered at baseline [52]. The BIQ consists of 19 items on 5-point bipolar scales, which display antithetical terms. The RSES consists of 10 statements assessing a set of feelings about self-esteem and self-acceptance; each statement is rated on a 4-point Likert scale ranging from 1 (“totally disagree”) to 4 (“totally agree”).

### Sample Size

In the study reported by Billhult et al [53], the mean relative improvement in CINV, as measured using a VAS, was 49.5% (SD 32.3%) in the placebo group and 73.5% (SD 32.2%) in the massage group. Assuming the same hypotheses, for a two-sided α risk of 5%, it was necessary to include 40 patients into each group to demonstrate a statistically significant difference between the two groups with a power of 90%.

### Randomization

Randomization was stratified by the type of cancer (ie, digestive or lung) and the presence or absence of metastases, with permuted blocks and random block sizes. It was performed by the Interactive Web Response System (version 7.5.720.1; Ennov Inc). Participants were enrolled by physicians at the Lyon Sud Hospital Centre thoracic and hepato-gastroenterology departments. Participants were allocated to the intervention group (ie, with foot reflexology) or to the control group (ie, without foot reflexology) before starting their treatment. Clinical research assistants generated the random allocation sequence and assigned participants to the intervention.

### Statistical Analysis

A detailed statistical analysis plan was written and validated before the data were unblinded. Initially, a linear model was considered to compare the variation in VAS points relative to acute nausea during the second cycle of chemotherapy between the two arms, adjusted by the type of cancer and by the presence or absence of metastases. Because of the low number of patients with nausea, we had to reconsider the statistical methods that were initially planned in the protocol to analyze the primary outcome. Instead of modeling the primary outcome, we compared the proportion of patients with an increase in VAS points of at least 2 between the two groups using the Fisher exact test. Statistical analyses of treatment effects were performed in the intention-to-treat (ITT) population for the primary endpoint, which included all randomized patients. Patients with missing acute nausea assessment during the second cycle of chemotherapy were considered as failure (VAS increase ≥2) in both treatment groups. Sensitivity analyses were performed by excluding patients without VAS assessments during the second cycle of chemotherapy (ie, per-protocol analysis). Other endpoints were analyzed on available data, without imputation of missing data (ie, patients lost to follow-up and questionnaires not completed or returned). Baseline clinical parameters were described using mean and SD or median and IQR for normally and nonnormally distributed continuous variables, respectively, and using frequency and percentage for categorical variables. Unless otherwise specified, categorical variables were compared between treatment groups using the Fisher exact test, and continuous variables were compared using the nonparametric Wilcoxon rank-sum test, with a two-sided *P* value of less than .05 being considered as statistically significant. All statistical analyses were performed using SAS software (version 9.4; SAS Institute Inc) in a Windows environment.

## Results

**Figure 3:**
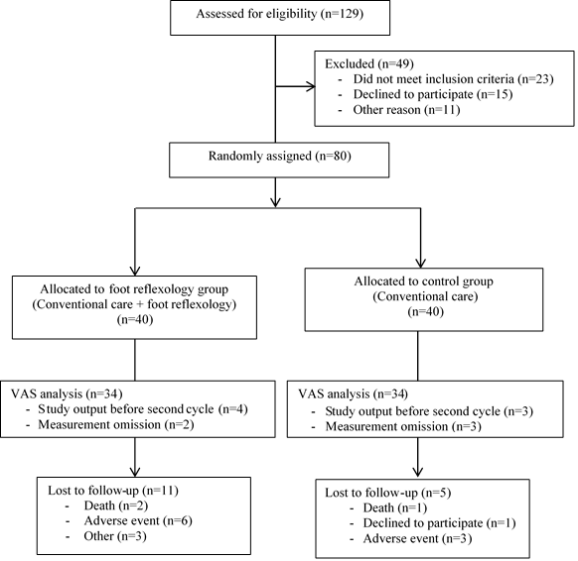
Modified CONSORT flow diagram for the individual randomized controlled trial REFYO-R of nonpharmacological treatment. REFYO-R: Reflexology/Yoga–Reflexology trial; VAS: visual analog scale.

A total of 80 patients were included and analyzed: 40 in the intervention group and 40 in the control group (Figure 3).

### Demographic and Clinical Characteristics

The majority of the participants in the foot reflexology and control groups were male. The mean age of the participants in the foot reflexology group was 63.4 (SD 11.5) years, and the mean age in the control group was 62.9 (SD 12.4) years. Most participants were diagnosed with lung cancer with metastasis and received moderately emetogenic chemotherapy (Table 1).

A total of 29 out of 40 (73%) participants in the foot reflexology group and 35 out of 40 (88%) participants in the control group received four cycles of chemotherapy (Table 2); 29 out of 40 (73%) patients in the foot reflexology group had their foot reflexology sessions at each cycle. The reasons for not performing the foot reflexology sessions were death, adverse events, and cancelled sessions owing to the COVID-19 pandemic.

**Table 1:** Characteristics of the study population (N=80).

Characteristic			Foot reflexology group (n=40)		Control group (n=40)
Sex (female), n (%)			13 (33)		17 (42)
Age in years, mean (SD)			63.4 (11.5)		62.9 (12.4)
Smoking, n (%)			14 (35)		6 (15)
**Diagnosis, n (%)**					
	Digestive cancer	16 (40)		17 (42)	
	Lung cancer	24 (60)		23 (57)	
Metastasis, n (%)		24 (60)		23 (57)	
**Type of chemotherapy (emetogenic level), n (%)**					
	Carboplatin (MEC^a^)	15 (37)		15 (37)	
	Oxaliplatin (MEC)	13 (32)		14 (35)	
	Cisplatin (HEC^b^)	12 (30)		11 (27)	

^a^MEC: moderately emetogenic chemotherapy.

^b^HEC: highly emetogenic chemotherapy.

**Table 2:** Chemotherapy cycles received by participants (N=80).

Number of cycles	Foot reflexology group (n=40), n (%)	Control group (n=40), n (%)	*P* value
1	3 (8)	3 (8)	.21
2	4 (10)	0 (0)	—^a^
3	4 (10)	2 (5)	—
4	29 (73)	35 (88)	—

^a^The *P* value for the entire group comparison is reported only in the top row.

### Efficacy Regarding CINV

Most participants in the foot reflexology (28/34, 82%) and control (32/34, 94%) groups had no nausea at the start of the second chemotherapy cycle. In the ITT analysis, where we considered all patients with missing assessments as having an increase of at least 2 VAS points, 6 out of 40 (15%) patients had an increase of at least 2 VAS points in the foot reflexology group compared with 13 out of 40 (33%) in the control group (*P*=.20). In the per-protocol analysis, there were significantly more patients with an increase of at least 2 VAS points among the control group (7/34, 21%; *P*=.001; Table 3).

A total of 22 out of 40 (55%) participants in the foot reflexology group and 29 out of 40 (73%) participants in the control group completed their daily diaries after at least one cycle. Regardless of the group, we observed that the incidence of delayed nausea was lower than delayed vomiting (Table 4). Across all cycles, there was a trend toward less frequent delayed nausea in the foot reflexology group (*P*=.28), a significantly less frequent consumption of antiemetic drugs (*P*=.04), and no significant difference in vomiting (*P*=.99; Table 4). There was a trend toward a perception of stronger severity for delayed nausea in the control group (*P*=.39; Table 5). Among 21 patients in the foot reflexology group who completed daily diaries and who answered the question (ie, “If you practiced self-massage, was it effective?”), 6 (29%) practiced self-massage and all considered it to be effective to decrease delayed nausea.

**Table 3:** Acute nausea during the second cycle of chemotherapy, as measured by the visual analog scale (VAS).

Measure	Foot reflexology group (n=34), n (%)	Control group (n=34), n (%)	*P* value
VAS1^a^ score >0	6 (18)	2 (6)	—^b^
VAS2^c^ score >0	4 (12)	8 (24)	—
VAS score increase ≥2	0 (0)	7 (21)	.001

^a^VAS1 is the VAS administered before the foot reflexology session for the intervention group and when the patient arrived at the outpatient or inpatient appointment for the control group.

^b^The *P* value concerns only the variation of the VAS score between VAS1 and VAS2 if ≥2.

^c^VAS2 is the VAS administered after the foot reflexology session for the intervention group and before leaving the hospital for the control group.

**Table 4:** Delayed nausea, delayed vomiting, and antiemetic drug use.

Outcome	Cycle 2, n (%)			Cycle 3, n (%)			Cycle 4, n (%)			End of study, n (%)			*P* value
	FR^a^ group (n=22)	Control group (n=29)	FR group (n=21)		Control group (n=28)	FR group (n=20)		Control group (n=26)	FR group (n=20)		Control group (n=25)		
Delayed nausea	11 (50)	18 (62)	9 (43)		17 (61)	7 (35)		15 (58)	7 (35)		12 (48)	.28	
Delayed vomiting	5 (23)	5 (17)	3 (14)		5 (18)	4 (20)		4 (15)	4 (20)		4 (16)	.99	
Antiemetic drug use	5 (23)	12 (41)	2 (10)		11 (39)	3 (15)		10 (38)	2 (10)		7 (28)	.04	

^a^FR: foot reflexology.

**Table 5:** Severity of delayed nausea between cycles of chemotherapy.

Severity	Cycle 2, n (%)		Cycle 3, n (%)		Cycle 4, n (%)		End of study, n (%)		*P* value
	FR^a^ group (n=9)	Control group (n=16)	FR group (n=9)	Control group (n=17)	FR group (n=7)	Control group (n=14)	FR group (n=7)	Control group (n=12)	
Very low to moderate	7 (78)	11 (69)	8 (89)	12 (71)	6 (86)	11 (79)	6 (86)	8 (67)	.39
Severe to unbearable	2 (22)	5 (31)	1 (11)	5 (29)	1 (14)	3 (21)	1 (14)	4 (33)	—^b^

^a^FR: foot reflexology.

^b^The *P* value for the entire group comparison is reported only in the top row.

### Efficacy Regarding Quality of Life and Anxiety

There was no significant difference in terms of quality of life (*P*=.32) or anxiety (*P*=.53) between the intervention and the control groups (Table 6).

**Table 6:** Quality of life (EORTC QLQ-C30) and anxiety (HADS) of the participants.

Measure		Baseline			End of study			*P* value
		Foot reflexology group (n=40)	Control group (n=40)	Foot reflexology group (n=40)		Control group (n=40)		
**EORTC-QLQ-C30^a^**								
	Participants, n (%)	36 (90)	36 (90)	27 (68)		33 (83)	—^b^	
	Score, mean (SD)	63.3 (14.6)	55.9 (11.4)	61.7 (15.4)		58.2 (12.4)	.32	
**HADS^c^**								
	Participants, n (%)	36 (90)	35 (88)	26 (65)		34 (85)	—	
	Score, mean (SD)	8.1 (3.4)	6.6 (3.5)	6.2 (2.5)		5.6 (3.85)	.53	

^a^EORTC QLQ-C30: European Organization for Research and Treatment of Cancer Quality of Life Questionnaire–Core 30.

^b^*P* values were only calculated for score comparisons.

^c^HADS: Hospital Anxiety and Depression Scale.

### Efficacy Regarding Self-esteem

At baseline, all patients reported having good self-esteem (RSES score >31); the median RSES score was 35 (IQR 32-38) for the control group among the 35 patients with assessment, and 33 (IQR 30-36.5) for the foot reflexology group among the 35 patients with assessment. At the end of the study, the average of BIQ score was 67.12 (SD 11.10) for the control group (25/40, 63%) and 59.76 (SD 10.15) for the foot reflexology group (17/40, 43%). After adjustment based on the initial RSES score and with a comparable RSES score, the average BIQ score decreased by 6.1 (95% CI –13.4 to –1.2) for the foot reflexology group compared to the control group (*P*=.10).

### Adverse Events

Adverse events were experienced by 12 participants: 7 (58%) participants in the foot reflexology group and 5 (42%) participants in the control group. Dyspnea, tinnitus, and leg-vein thrombosis were experienced by participants in the foot reflexology group only. Sepsis, neutropenia, and pulmonary embolism were experienced by participants in the control group only. Renal failure and radiation esophagitis were experienced by participants in both groups. None of the adverse events were attributed to foot reflexology, according to the physicians.

## Discussion

### Principal Findings

The main objective of this study was to assess the benefits of foot reflexology in acute CINV. More than half of the participants were men with metastatic lung cancer, with an average age of 63 years, who received moderately emetogenic chemotherapy. These results, which included both male and female patients, showed that foot reflexology significantly decreased acute nausea in patients with lung or digestive cancer who were receiving chemotherapy. These results confirm those of previous studies that included only female patients and that provided only a low level of evidence [36,37].

Among the secondary objectives, we assessed the benefits of foot reflexology in terms of the frequency of delayed CINV, because no study published to date has assessed this outcome. Regarding the frequency of delayed vomiting, foot reflexology did not show any benefit. Regarding the frequency of delayed nausea, we observed that patients in the foot reflexology group tended to have less delayed nausea. We can assume that the benefits of foot reflexology observed in acute nausea contributed to better control of delayed nausea, resulting in a decrease in its severity; in fact, Schnell [54] has shown that effective prevention and control of acute CINV significantly reduced the risk of delayed symptoms in the same cycle. We also assessed the perception of the severity of delayed CINV, because taking into account the subjective points of view of patients contributes to the improvement of the management of treatment toxicities [55]. Regarding the perception of the severity of delayed CINV, patients in the control and foot reflexology groups reported it as more severe than in Morin et al’s survey [19]. One of the objectives of this survey was to assess the differences in perception of the incidence and impact of CINV and radiotherapy-induced vomiting between health care professionals and patients. In that study [19], 12% of the patients reported that their delayed CINV was severe. The difference with the results in this study may be explained by the fact that Morin et al’s survey included patients with cancer who had chemotherapy in the last 24 months, which may have led to memory bias; furthermore, that survey did not indicate the type of chemotherapy patients received. Regarding the perception of the severity of delayed nausea in this study in particular, patients in the foot reflexology group expressed lower severity with a decreasing trend between the first and fourth chemotherapy treatment. Lastly, although vomiting is better controlled, delayed nausea remains a significant problem in practice [16]. Several factors contribute to the suboptimal management of delayed nausea, such as health care professionals’ underestimation of their severity and nonadherence to antiemetic regimens [16]; patients reported nonadherence, particularly because they were already taking several pills, and they reported that CINV was accepted as an inevitable side effect of treatment [19]. However, nausea has a negative impact on patients’ quality of life [12]. This is why the foot reflexology group was taught self-massage to relieve their CINV in a nonmedicinal way, if they desired. The 29% of patients who practiced self-massage all reported that it was effective. Moreover, we observed in the foot reflexology group that the consumption of antiemetic drugs between each cycle was significantly lower. In consideration of these results, we can suggest that self-massage seems to be a promising complementary care treatment to standard antiemetic treatment to improve the management of delayed nausea. We could also consider involving family caregivers. In fact, Stephenson et al [34] have shown that foot reflexology practiced by family caregivers significantly reduced pain and anxiety in patients with metastases, while promoting social connections.

Overall, irrespective of the group, we observed that the occurrence of acute and delayed nausea was more frequent than vomiting, as has also been reported in previous studies [9,10,18,19,56,57]. Nevertheless, the results of this study demonstrated that acute nausea was lower than in those studies. Among risk factors, sex of participants is a predictive value in the development of CINV [10], and we observed a high representation of males in our study. On another note, since previous studies were conducted before 2016, we can assume that new antiemetic drugs, specifically the fixed-combination drug netupitant/palonosetron (NEPA) and rolapitant, which were marketed after 2017, are more effective for acute nausea [8,13].

In France, an update of the AFSOS (Association Francophone des Soins Oncologiques de Support) standard for nausea and vomiting induced by cancer treatments was also made in 2018 [13]. According to these guidelines, acupuncture and the treatment of anxiety with psychotropic drugs in association with, or alternatively to, nondrug practices (meditation, relaxation, hypnosis, etc) and cannabinoids, in addition to conventional antiemetic drug prophylaxis, may also prove effective but are in need of further investigation [8,13]. The results of this study may suggest that foot reflexology could be added to these guidelines in the future.

In contrast, foot reflexology did not have a significant effect on quality of life and anxiety, unlike findings reported in previous studies [32-35]. However, three of those previous studies [32,34,35] were conducted using pre- and postinterventions and suggested that the efficacy of foot reflexology had short-term effects. Furthermore, the Sharp et al study [33] demonstrated a significant effect on quality of life in patients with breast cancer. Patients received a single 1-hour session weekly for 8 weeks. We can, thus, suggest that the number of sessions was insufficient to demonstrate a benefit in terms of quality of life in this study. Even if no significant effect on anxiety was found, we observed a decrease in the anxiety score in both groups between baseline and the end of the study. This may be due to the effectiveness of the psychological support that was offered to all patients, as the Sharp et al study highlighted [33]. Finally, we can also question whether the HADS was the most appropriate scale to use. In fact, a recent study has underlined that the HADS is quicker in terms of administration and scoring when using in oncology settings than the two gold-standard tools (ie, the STAI-S [State-Trait Anxiety Inventory–State] and the CES-D [Center for Epidemiological Studies–Depression]) that were employed but presents more false positives [58].

### Limitations

This study had some limitations. First, patient recruitment was only done at one cancer center, so the results are not representative of the general population; a larger study would ensure that the results are generalizable. Second, the number of subjects necessary to assess the primary endpoint was not reached because few patients had acute nausea at cycle 2; however, the benefits of reflexology were demonstrated, as the results were significant. Moreover, few patients completed the BIQ, questions of which were not cancer specific and may not have been adapted to patients with cancer; semistructured interviews seem more appropriate to assess these outcomes. Lastly, some patients did not complete their daily diary. To best assess delayed nausea, we should consider calling the patient within 5 days of hospital discharge after each cycle.

### Conclusions

In conclusion, according to the results of this study, foot reflexology significantly decreased acute nausea with significantly less consumption of antiemetic drugs between each cycle among patients with lung or digestive cancer. We also observed a lower occurrence of delayed nausea in the reflexology group. Therefore, foot reflexology seems to be a promising and innovative complementary treatment to conventional antiemetic drugs. To assess the performance of this intervention in routine practice, a larger study with several health care centers would be relevant with a cluster RCT. We also plan to investigate the relationship between nausea and vomiting and foot reflexology at the cerebral level using functional magnetic resonance imaging.

## Appendix

Multimedia Appendix 1 2017 CONSORT checklist of information to include when reporting a randomized trial assessing nonpharmacologic treatments (NPTs) - REFYO-R.

## Abbreviations

AFSOS: Association Francophone des Soins Oncologiques de Support
APICIL: Association de Prévoyance Interprofessionnelle des Cadres et Ingénieurs de la région Lyonnaise
BIQ: Body Image Questionnaire
CAM: complementary and alternative medicine
CES-D: Center for Epidemiological Studies–Depression
CINV: chemotherapy-induced nausea and vomiting
DRCI: Direction de la Recherche Clinique et de l’Innovation
EORTC QLQ-C30: European Organization for Research and Treatment of Cancer Quality of Life Questionnaire–Core 30
HADS: Hospital Anxiety and Depression Scale
HADS-A: Hospital Anxiety and Depression Scale–Anxiety
HADS-D: Hospital Anxiety and Depression Scale–Depression
ITT: intention-to-treat
NEPA: netupitant/palonosetron
RCT: randomized controlled trial
REFYO-R: Reflexology/Yoga–Reflexology trial
RSES: Rosenberg Self-Esteem Scale
STAI-S: State-Trait Anxiety Inventory–State
VAS: visual analog scale
