# Supplementary material for: The Effect of Foot Reflexology on Chemotherapy-Induced Nausea and Vomiting in Patients With Digestive or Lung Cancer: Randomized Controlled Trial
Source: JMIR Cancer. 2021 Nov 5;7(4):e25648. doi: 10.2196/25648 (PMC8663669; doi:10.2196/25648)
Supplement: Multimedia Appendix 1 [file cancer_v7i4e25648_app1.pdf]

2017 CONSORT checklist of information to include when reporting a randomized trial assessing nonpharmacologic treatments (NPTs)*. Modifications of the extension appear in italics and blue.

Section/Topic Item	Checklist item no.	CONSORT item	Extension for NPT trials
**Title and abstract**			
	1a	**Identification as a randomized trial in the title**<br>The effect of foot reflexology on chemotherapy-induced nausea and vomiting in digestive or lung cancer patients: a randomized controlled trial	

Cite as: *Boutron I, Altman DG, Moher D, Schulz KF, Ravaud P. CONSORT Statement for Randomized Trials of Nonpharmacologic Treatments: A 2017 Update and a CONSORT Extension for Nonpharmacologic Trial Abstracts. Annals of Internal Medicine. 2017 Jul 4;167(1):40–7.*

Section/Topic Item	Checklist item no.	CONSORT item	Extension for NPT trials
	1b	**Structured summary of trial design, methods, results, and conclusions (for specific guidance see CONSORT for abstracts)**<br>**Background**<br>Cancer is a chronic disease with an incident worldwide had been 24.5 million and 9.6 million deaths in 2017. Lung and colorectal cancer are the most common cancer for both sexes and according to national and international recommendations platinum-based chemotherapy is the reference adjuvant treatment. This chemotherapy can be moderately to highly emetogenic. Despite antiemetic therapy, chemotherapy-induced nausea and vomiting may persist. Moreover, cancer patient are increasingly interested in alternative and complementary medicines and express the desire that non-pharmacological treatments be used in hospitals. Among alternative and complementary medicines, foot reflexology decreases significantly the severity of chemotherapy-induced nausea and vomiting in breast cancer patients.<br>**Objectives**<br>The primary objective of the present study was to assess the benefits of foot reflexology as a complement to conventional treatments on severity of acute chemotherapy-induced nausea and vomiting in digestive or lung cancer patients. The secondary objectives assessed were the frequency and severity of delayed chemotherapy-induced nausea and vomiting, quality of life, anxiety, and self-esteem.<br>**Methods**<br>The present study was conducted between April 2018 and April 2020 in French University Hospital. This is an open-label randomized controlled trial. Participants are randomized into two groups: 40 to interventional group (conventional care with foot reflexology) and 40 to control group (conventional care without foot reflexology). Foot reflexology sessions (30 minutes) are performed on an outpatient or inpatient. Eligible participants are patients with a lung or digestive cancer with indication for platinum-based chemotherapy.<br>**Results**<br>The severity of acute nausea and vomiting was assessed with a visual analogue scale during the second cycle of chemotherapy. A significant increase of at least 2 points was observed for control group (20.6%, P = 0.01). Across all cycle, the foot reflexology group showed a trend towards less frequent delayed nausea (P=0.28), a significantly less frequent consumption of antiemetic drugs (P=0.04), and no significant difference for vomiting (P=0.99); there was a trend towards a perception of stronger severity for delayed nausea in the control group (P=0.39). According to quality of life and anxiety, there was no significant difference between the interventional group and the control group (P=0.32 and P=0.53 respectively).<br>**Conclusions**<br>In conclusion, the present study results indicated that foot reflexology decreased significantly the severity of acute nausea and consumption of antiemetic drugs in lung and digestive cancer patients. No side effects from foot reflexology have been noted. In order to better respond to a desire of patients for non-pharmacological treatments and CAMs to be used in hospitals to improve their care, the results of this study showed that foot reflexology seems to be a promising complement to conventional antiemetic drugs. To assess the performance of this intervention in routine practice, a larger study with several health care centers would be relevant with a cluster RCT.<br>**Trial registration**<br>The present study registered with *clinicaltrials.gov*: NCT03508180 (28/06/2018)<br>**Funding**<br>APICIL's foundation funds reflexologists who are involved in this study.	*Refer to CONSORT extension for abstracts for NPT trials*<br>*YES*
**Introduction**			

Section/Topic Item	Checklist item no.	CONSORT item	Extension for NPT trials
Background and objectives	2a	**Scientific background and explanation of rationale**<br>Cancer patients are increasingly interested in complementary and alternative medicines (CAMs) [1]. According to the systematic review reported by Keen *et al*. the main reasons why patients use CAMs is to treat their cancer, to treat side effects of treatment, and to improve quality of life [2]. Patients with chronic disease, including cancer, express the desire that non-pharmacological treatments and CAMs be used in hospitals [3]. At the time of writing, the most frequently provided CAMs in private and public oncology centres in European countries are mind-body techniques, acupuncture, homeopathy, energy therapies, health promotion, traditional herbal medicine, as well as manipulation and body-based practices (kinesiology, osteopathy, physiotherapy, and reflexology) [4]. Foot reflexology is a holistic approach that is reported to decrease significantly the severity of anxiety in patients with metastatic cancer [5] and improves the perceived pain and anxiety [6]. Moreover, a significant decrease in the severity of chemotherapy-induced nausea and vomiting (CINV) has been observed in breast cancer patients receiving chemotherapy [7]. So foot reflexology seems very interesting because among the side effects induced by chemotherapy, CINV is the most feared by patients [8]; it decreases overall quality of life [9,10] and induces metabolic complications [8]. In addition CINV can lead to dose reduction, postponement of treatment and even discontinuation [11] which can decrease the effectiveness of treatment [12]. To anticipate or relieve CINV, the recommendation is to prescribe antiemetic drugs [8], but some patients report that they are not sufficiently effective [13]. According to national and international recommendations, adjuvant treatment for lung and digestive cancers, which are among the most frequent in the world [14], is chemotherapy most of which are moderately to highly emitting [15–19]. The hypothesis is that foot reflexology delivered at each course of chemotherapy decreases the severity of CINV and anxiety and improves quality of life.	
	2b	**Specific objectives or hypotheses**<br>The aim of the present study is therefore to assess foot reflexology as a CAM to decrease the side effects induced by chemotherapy, specifically CINV by platinum-based chemotherapy, in patients with lung or digestive cancer.	
**Methods**			
Trial design	3a	**Description of trial design (such as parallel, factorial) including allocation ratio**<br>Open label randomised clinical trial (RCT) in which the patients are randomised to two groups at a ratio of 1:1;	When applicable, how care providers were allocated to each trial group<br>NA
	3b	Important changes to methods after trial commencement (such as eligibility criteria), with reasons<br>NA	
Participants	4a	**Eligibility criteria for participants**<br>age ≥ 18 years; patients with a lung or digestive cancer with indication of management with platinum-based chemotherapy; WHO performance status ≤ 2; patients affiliated to the national social security system or equivalent; patients able to complete the questionnaires (comprehension of oral and written French language); and written informed consent.	When applicable, eligibility criteria for centers and for *care providers*<br>NA
	4b	**Settings and locations where the data were collected**<br>All data are collected from outpatients or inpatients	

Section/Topic Item	Checklist item no.	CONSORT item	Extension for NPT trials
Interventions†	5	The interventions for each group with sufficient details to allow replication, including how and when they were actually administered<br><br>The patients randomised to the interventional group will benefit from a foot reflexology session (30 minutes) at each chemotherapy course for four courses. Foot reflexology will be administered by two qualified reflexologists (they have been trained in the French school *École des Techniques en Réflexologie*). All patients will continue to receive standard antiemetic treatments.<br><br>According to Lee's meta-analysis the optimal comparator is a control-group with conventional care without foot reflexology or massage therapy [20].	**Precise details of both the experimental treatment and comparator**
	5a	Foot reflexology is CAM based on the principle of acupressure that helps the body to restore homeostasis. It is a holistic approach which allows one to apprehend the body as a whole. Each part of the body is represented by a zone or reflex point on the foot. The reflexologist stimulates each reflex zone using specific thumb and finger techniques on the patient's feet. Depending on the objective to be achieved, the zones on the feet are stimulated using different types of pressure. During a session focused on the treatment of CINV, the reflexologist mainly stimulates the reflex points related to the digestive system, as well as the lymphatic and kidney zones to help the body eliminate toxins. The reflexology chart used in the present clinical study is based on the one proposed by Eunice Ingham [21].<br>The reflex zones of the whole body are equally found on the hands; the reflexologist shows the patient the appropriate zones during the first reflexology session so that he/she can stimulate these reflex points between	**Description of the different components of the interventions and, when applicable, description of the procedure for tailoring the interventions to individual participants**.<br>sessions.
	5b	Protocol of intervention was standardized by reflexologists. During a session focused on the treatment of CINV, the reflexologist mainly stimulates the reflex points related to the digestive system, as well as the lymphatic and kidney zones to help the body eliminate toxins. The reflexology chart used in the present clinical study is based on the one proposed by Eunice Ingham [21]	**Details *of whether and* how the interventions were standardized.**<br>.
	5c.		Details *of whether and* how adherence of care providers to the protocol was assessed or enhanced<br>NA
	5d	*Adherence of participants to reflexology is assessed with a follow-up logbook completed between each course of treatment*	*Details of whether and how adherence of participants to interventions was assessed or enhanced*

Section/Topic Item	Checklist item no.	CONSORT item	Extension for NPT trials
Outcomes	6a	Completely defined pre-specified primary and secondary outcome measures, including how and when they were assessed<br>**Primary outcome**<br>**Nausea/vomiting**<br>The primary endpoint is the relative change in the severity of nausea and vomiting, as assessed by a VAS (subjective assessment of the severity of nausea: 0-mm no symptom 100-mm paroxysm of nausea or vomiting). For patients in the interventional group, this is measured when the patient is arrives at the outpatient clinic and after the foot reflexology session during the second course of chemotherapy. For patients in the control group, this is measured when the patient arrives at the outpatient clinic and after the second course of chemotherapy. The assessment is carried out by a nurse or clinical research assistant.<br>**Secondary outcomes**<br>**Nausea/vomiting**<br>The benefits of foot reflexology on CINV will also be assessed using the proportion of between chemotherapy courses during which the patient took at least one antiemetic drug and by the frequency of CINV.<br>**Quality of life**:<br>The benefits of foot reflexology on quality of life will be assessed by the relative change in the overall European Organisation for Research and Treatment of Cancer Quality of Life Questionnaire – Core 30 items EORTC QLQ C30 [22] score between the end of study visit and the first administration of chemotherapy.<br>**Anxiety:**<br>The benefits of foot reflexology on anxiety will be assessed by the relative change in the overall hospital and anxiety depression scale (HADS) [23] score between the end of study visit and the first administration of chemotherapy.<br>**Body image**:<br>The benefits of foot reflexology at the level of body image will be assessed at the end of study visit using the body image questionnaire (BIQ) [24] which measures body image at a given time; the analysis of the BIQ takes into account the self-esteem assessed at inclusion using the Rosenberg scale [25].	
	6b	Any changes to trial outcomes after the trial commenced, with reasons	
Sample size	7a	**How sample size was determined**<br>In the study reported by Billhult *et al.*, the mean improvement for CINV (measured using a visual analogue scale, VAS) in the placebo group was 49.5%, and 73.5% in the foot reflexology group (with a common standard deviation of 32.2%) [26]. Assuming these same hypotheses, for a two-sided alpha risk of 5%, it is necessary to include 40 patients per group to demonstrate a statistically significant difference between the two groups with a power of 90%.	When applicable, details of whether and how the clustering by care providers or centers was addressed<br>NA
	7b	When applicable, explanation of any interim analyses and stopping guidelines<br>NA	
Randomization:			
- Sequence generation	8a	**Method used to generate the random allocation sequence**<br>The randomisation procedure is performed by Interactive Web Response System (IWRS) via ClinSight software (Ennov Clinical version 7.5.710. groupe Ennov France)	

Section/Topic Item	Checklist item no.	CONSORT item	Extension for NPT trials
	8b	**Type of randomization; details of any restriction (such as blocking and block size)**<br>Randomisation 1:1	
- Allocation concealment mechanism	9	Mechanism used to implement the random allocation sequence (such as sequentially numbered containers), describing any steps taken to conceal the sequence until interventions were assigned	
- Implementation	10	**Who generated the random allocation sequence, who enrolled participants, and who assigned participants to interventions**<br>Physicians enroll participants. Nurse or clinical research assistant generate the random allocation sequence and assign participants to intervention	
Blinding	11a	If done, who was blinded after assignment to interventions (for example, participants, care providers, those assessing outcomes) and how<br><br>NA	~~Whether or not those administering co-interventions were blinded to group assignment~~<br>If done, who was blinded after assignment to interventions (e.g., participants, care providers, *those administering co-interventions,* those assessing outcomes) and how
	11b	If relevant, description of the similarity of interventions<br>NA	~~If blinded, method of blinding and description of the similarity of interventions~~
	11c	*According to Lee's meta-analysis the optimal comparator is a control-group with conventional care without foot reflexology or massage therapy (22). To limit bias, conventional care is similar for all patients with standard antiemetic drugs.*	***If blinding was not possible, description of any attempts to limit bias***

Cite as: *Boutron I, Altman DG, Moher D, Schulz KF, Ravaud P. CONSORT Statement for Randomized Trials of Nonpharmacologic Treatments: A 2017 Update and a CONSORT Extension for Nonpharmacologic Trial Abstracts. Annals of Internal Medicine. 2017 Jul 4;167(1):40–7.*

Section/Topic Item	Checklist item no.	CONSORT item	Extension for NPT trials
Statistical methods	12a	**Statistical methods used to compare groups for primary and secondary outcomes**<br>A detailed statistical analysis plan was written and validated before the data were unblinded. Because of the low number of patients with nausea, we had to reconsider the statistical methods that were initially planned in the protocol to analyze the primary outcome. Instead of modelling the primary outcome, we compared the proportion of patients with at least 2 or more increased unit on the VAS between the two groups using Fisher's exact test. Statistical analyses of treatment effects were performed in the intention-to-treat (ITT) population, which included all randomized patients with available information. Baseline clinical parameters were described using mean and standard deviation (SD), or median and interquartile ranges [IQR] for normally and non-normally distributed continuous variables, respectively, and with frequency and percentages for categorical variables. Unless otherwise specified, categorical variables were compared between treatment groups using Fisher's exact test, and continuous variables with the non-parametric Wilcoxon rank-sum test, with a two-sided P value of less than 0.05 being considered as statistically significant. All statistical analyses were performed using the SAS® Software version 9.4 in a Windows environment (SAS institute, Cary, NC, US).	When applicable, details of whether and how the clustering by care providers or centers was addressed
	12b	**Methods for additional analyses, such as subgroup analyses and adjusted analyses**<br>For the primary endpoint, a secondary analysis will be performed per protocol, including patients with the endpoint assessment, and for whom the strategy allocated during randomisation was fully implemented (patients allocated to the foot reflexology group but not having received their four sessions of foot reflexology will be excluded from the analysis).	
**Results: Enrollment currently ongoing**			
Participant flow (a diagram is strongly recommended **SEE last page**)	13a	For each group, the numbers of participants who were randomly assigned, received intended treatment, and were analyzed for the primary outcome	The number of care providers or centers performing the intervention in each group and the number of patients treated by each care provider or in each center
	13b	For each group, losses and exclusions after randomization, together with reasons<br>Excluded (n=49)<br>• Did not meet inclusion criteria (n=23)<br>• Declined to participate (n=15)<br>• Other reason (n=11)	
	13c	+/- 2 days	*For each group, the delay between randomization and the initiation of the intervention*

Section/Topic Item	Checklist item no.	CONSORT item	Extension for NPT trials
	new	The patients randomized to the interventional group (N=40) received 4 foot reflexology sessions (30 minutes) during chemotherapy infusion every 2 or 3 weeks (according to the chemotherapy protocol). They were administered by three qualified reflexologists. The reflexology chart used in the present clinical study is based on the one proposed by Eunice Ingham [43]. Intervention was standardized: to calm nausea and vomiting, the upper and lower digestive reflex points, as well as the metabolism smooth muscles reflex points (lymphatic system gently, kidneys + bladder, lungs, thyroid and para-thyroid) were stimulated. To provide deep relaxation to target anxiety, the diencephalon reflex points, scapular belt reflex points, reflex points of the diaphragm, and reflex points of the spine were stimulated. After each stimulated reflex points relaxation movements [43]. During the first reflexology session the reflexologist trained the patients of the foot reflexology group only the appropriate zones on the hands to relieve nausea.	Details of the experimental treatment and comparator as they were implemented
Recruitment	14a	Dates defining the periods of recruitment and follow-up<br>**This study is an open label randomised controlled trial conducted over 22 months (18 months intervention and 4 months follow-up). This study started 06.14.2018 and ended 04/08/2020**	
	14b	Why the trial ended or was stopped<br>**80 patients enrolled**	
Baseline data	15	A table showing baseline demographic and clinical characteristics for each group (see table below)	When applicable, a description of care providers (case volume, qualification, expertise, etc.) and centers (volume) in each group.

	Foot reflexology (n=40)	Control (n=40)
Sex – Female n (%)	13 (32.5)	17 (42.5)
Mean age, years (SD)	63.4 (11.5)	62.9 (12.4)
Tabaco, n (%)	14 (35.0)	6 (15.0)
Diagnosis, n (%)		
Digestive cancer	16 (40.0)	17 (42.5)
Lung cancer	24 (60.0)	23 (57.5)
Metastasis, n (%)	24 (60.0)	23 (57.5)
Chemotherapy with, n (%)		
Carboplatin (MEC)	15 (37.8)	15 (37.5)
Oxaliplatin (MEC)	13 (32.2)	14 (35.0)
Cisplatin (HEC)	12 (30.0)	11 (27.5)

Abbreviations: SD, standard deviation; MEC, moderately emetogenic chemotherapy; HEC, highly emetogenic chemotherapy

Section/Topic Item	Checklist item no.	CONSORT item	Extension for NPT trials
Numbers analyzed	16	For each group, number of participants (denominator) included in each analysis and whether the analysis was by original assigned groups<br>**40 per group**	

<table>
<tr><th>Section/Topic Item</th><th>Checklist item no.</th><th>CONSORT item</th><th>Extension for NPT trials</th></tr>
<tr><td>Outcomes and estimation</td><td>17a</td><td>For each primary and secondary outcome, results for each group, and the estimated effect size and its precision (such as 95% confidence interval)

Most participants in the foot reflexology (82.4%) and control groups (94.1%) had no nausea at the start of the second chemotherapy cycle. There was significantly more frequently an increase of at least 2 points among the control group (20.6%, P=0.011; Table 3).

Table 3: Acute nausea during the second cycle of chemotherapy
<table>
<tr><th></th><th>Foot reflexology (n=34)</th><th>Control (n=34)</th><th>P</th></tr>
<tr><td>VAS1 >0, n (%)</td><td>6 (17.5)</td><td>2 (5.9)</td><td></td></tr>
<tr><td>VAS2 >0, n (%)</td><td>4 (11.8)</td><td>8 (23.5)</td><td></td></tr>
<tr><td>VAS increase ≥2, n (%)</td><td>0 (0.0)</td><td>7 (20.6)</td><td>0.0011</td></tr>
</table>
Abbreviations: VAS, visual analogic scale

Twenty-two (52.5%) of the foot reflexology group and 29 (72.5%) of the control group completed their daily diaries after at least one cycle. Whatever the group, we observed that incidence of delayed nausea was lower than delayed vomiting (Table 4). Across all cycles, there was a trend towards less frequent delayed nausea in the foot reflexology group (P=0.28), a significantly less frequent consumption of antiemetic drugs (P=0.037), and no significant difference for vomiting (P=0.99); there was a trend towards a perception of stronger severity for delayed nausea in the control group (P=0.39). Among the 21/22 patients in the foot reflexology group who completed daily diaries and answered the question, 6 (28.6%) practiced self-massage and all considered it to be effective to decrease delayed nausea.

There was no significant difference in terms of quality of life (P=0.32) or anxiety (P=0.53) between the interventional and the control groups.

At the baseline, self-esteem is low irrespective of the group (mean of score<31); the mean of score RSES was 23.6 (SD 2.7) for control group (n=35) and 25.3 (SD 2.7) for foot reflexology group (n=35; P<0.004). At the end of study, the mean of score BIQ was 51.1 (SD 20.4) for control group (n=22) and 41.1 (SD 17.8) for foot reflexology group (n=16; P=0.15).</td><td></td></tr>
<tr><td></td><td>17b</td><td>For binary outcomes, presentation of both absolute and relative effect sizes is recommended</td><td></td></tr>
<tr><td>Ancillary analyses</td><td>18</td><td>Results of any other analyses performed, including subgroup analyses and adjusted analyses, distinguishing pre-specified from exploratory
NA</td><td></td></tr>
<tr><td>Harms</td><td>19</td><td>All important harms or unintended effects in each group (for specific guidance see CONSORT for harms)
Adverse events were experienced by 12 participants (7 foot reflexology group, FR and 5 control group, C): dyspnea (FR), tinnitus (FR), sepsis (C), neutropenia (C), renal failure (FR and C), leg-vein thrombosis (FR), radiation esophagitis (FR and C), and pulmonary embolism (C). None of the adverse events were attributed to foot reflexology by the physicians.</td><td></td></tr>
</table>

Section/Topic Item	Checklist item no.	CONSORT item	Extension for NPT trials
**Discussion**			
Limitations	20	**Trial limitations, addressing sources of potential bias, imprecision, and, if relevant, multiplicity of analyses**<br>The present study had some limitations. First, patient's recruitment was only done at one cancer center the results are not representative therefore of the general population; a larger study would ensure that the results are generalizable. Second, the number of subjects necessary to assess the primary endpoint is not reached because few patients had acute nausea at cycle 2; however, the benefits of reflexology are demonstrated as the results are significant. Moreover, few patients completed the BIQ that are not cancer-specific and may not have been adapted to such patients; semi-structured interviews seem more appropriate to assess these outcomes. Lastly, some patients did not complete their daily diary. To best assess delayed nausea, we should consider calling the patient within 5 days of hospital discharge after each cycle.	In addition, take into account the choice of the comparator, lack of or partial blinding, and unequal expertise of care providers or centers in each group
Generalizability	21	**Generalizability (external validity, applicability) of the trial findings**<br><br>The main objective of the present study was to assess the benefits of foot reflexology in acute CINV. More than half of the participants were men with metastatic lung cancer with an average age of 63 years who received moderately emetogenic chemotherapy. The present results that included both male and female patients showed that foot reflexology decreased significantly acute nausea in lung and digestive cancer patients receiving chemotherapy. These results confirm those of previous studies that included only female patients, and that provided only a low level of evidence [35], [36].<br>A larger study with several centres this would be relevant to ensure that the results are generalizable.	**Generalizability (external validity) of the trial findings according to the intervention, comparators, patients, and care providers and centers involved in the trial**<br>conventional cares are similar within the various private and public health care centres in France, so this is a good comparator for generalizability
Interpretation	22	**Interpretation consistent with results, balancing benefits and harms, and considering other relevant evidence**<br>In conclusion, according to the results of this study foot reflexology decreased significantly acute nausea with a significant less consumption of antiemetic drug between each cycle in lung and digestive cancer patients. We also observed a lower occurrence of delayed nausea in the reflexology group. Therefore, foot reflexology seems to be a promising and innovative complement to conventional antiemetic drugs. To assess the performance of this intervention in routine practice, a larger study with several health care centres would be relevant with a cluster RCT.	
**Other information**			
Registration	23	**Registration number and name of trial registry**<br>The present study registered with *clinicaltrials.gov*: NCT03508180 (28/06/2018)	
Protocol	24	**Where the full trial protocol can be accessed, if available**<br>***(JMIR Res Protoc 2020;0(0):e0)*** doi: 10.2196/17232	
Funding	25	**Sources of funding and other support (such as supply of drugs), role of funders**<br>APICIL's foundation funds reflexologists who are involved in this study	

**Additions or modifications to the 2010 CONSORT checklist. CONSORT = Consolidated Standards of Reporting Trials*
†The items 5, 5a, 5b, 5c, 5d are consistent with the Template for Intervention Description and Replication (TIDieR) checklist

SHEMATIC DIAGRAM: Time schedule of enrolment, interventions (including any run-ins and washouts), assessments, and visits for participants

<table>
<tr><th>STEPS</th><th>V1
Pre-screening</th><th>V2
Inclusion/randomisation</th><th>V3</th><th>V4</th><th>V5</th><th>V6
End of study</th></tr>
<tr><th>Moment
Actions</th><th>J-30 à J-15
consultation</th><th>C1
J0 +/-1j</th><th>C2
J14-J21* +/-1j</th><th>C3
J28-J42* +/-1j</th><th>C4
J42-J63* +/-1j</th><th>C4 +/- 15 j</th></tr>
<tr><td>Patient's information</td><td>X</td><td></td><td></td><td></td><td></td><td></td></tr>
<tr><td>Signed Informed Consent</td><td>X</td><td>X</td><td></td><td></td><td></td><td></td></tr>
<tr><td>Inclusion</td><td></td><td>X</td><td></td><td></td><td></td><td></td></tr>
<tr><td>Randomisation</td><td></td><td>X</td><td></td><td></td><td></td><td></td></tr>
<tr><td>Clinical examination[1]</td><td>X</td><td>X</td><td>X</td><td>X</td><td>X</td><td>X</td></tr>
<tr><td>Foot reflexology (only interventional group)</td><td></td><td colspan="4">X</td><td></td></tr>
<tr><td>VAS (nausea/vomiting)</td><td></td><td></td><td>X</td><td></td><td></td><td></td></tr>
<tr><td>EORTC QLQ-C30</td><td></td><td>X</td><td></td><td></td><td></td><td>X</td></tr>
<tr><td>HADS</td><td></td><td>X</td><td></td><td></td><td></td><td>X</td></tr>
<tr><td>Rosenberg' scale</td><td></td><td>X</td><td></td><td></td><td></td><td></td></tr>
<tr><td>QIC</td><td></td><td></td><td></td><td></td><td></td><td>X</td></tr>
<tr><td>Nausea/vomiting diary completed between each course of chemotherapy</td><td></td><td colspan="4">X</td><td></td></tr>
<tr><td>Collection AE/SAE</td><td></td><td>X</td><td>X</td><td>X</td><td>X</td><td>X</td></tr>
<tr><td>Collection concomitant treatments[2]</td><td></td><td>X</td><td>X</td><td>X</td><td>X</td><td>X</td></tr>
</table>

[1] weight, blood pressure and WHO performance status

[2] http://www.afsos.org/fiche-referentiel/nausees-vomissements-chimio-induits/

** According to chemotherapy protocol (every 14 days or 21 days)*

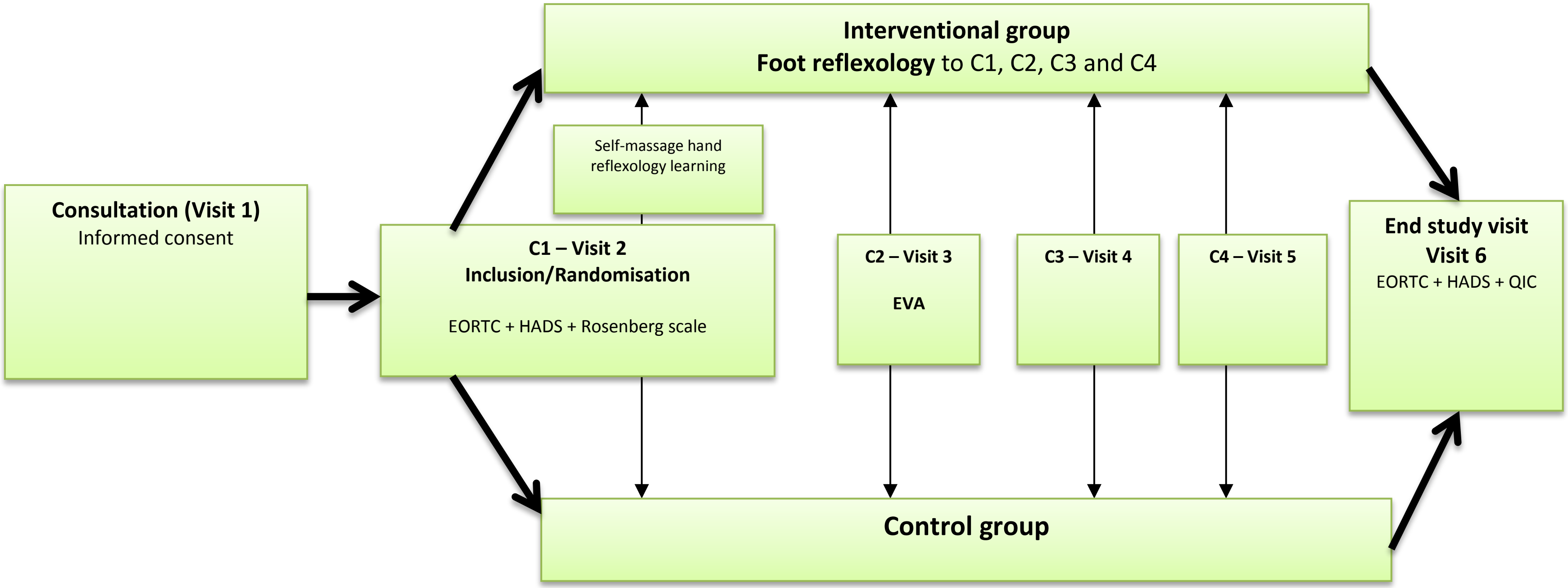

Cite as: *Boutron I, Altman DG, Moher D, Schulz KF, Ravaud P. CONSORT Statement for Randomized Trials of Nonpharmacologic Treatments: A 2017 Update and a CONSORT Extension for Nonpharmacologic Trial Abstracts. Annals of Internal Medicine. 2017 Jul 4;167(1):40–7.*

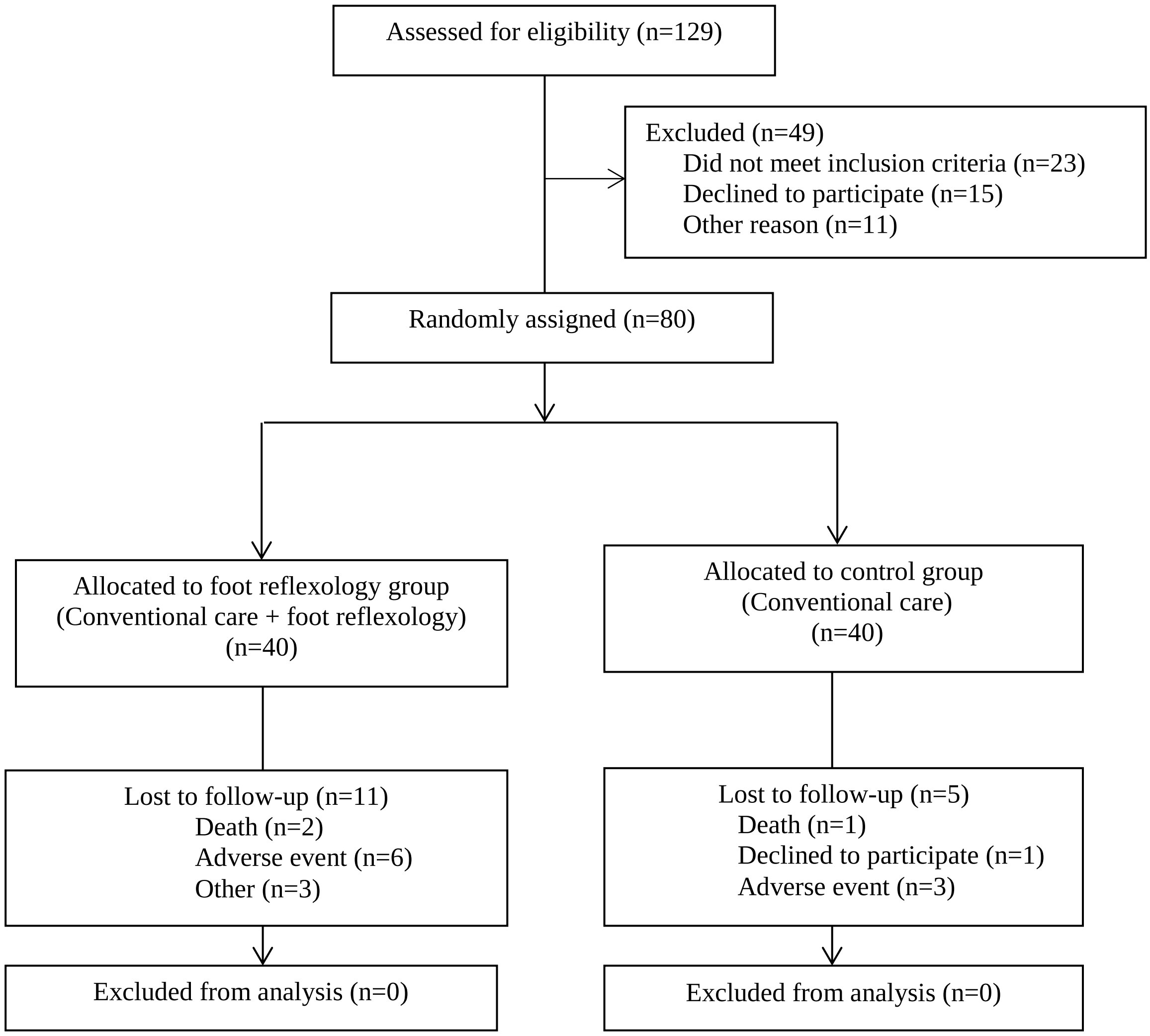

FLOW DIAGRAM: Modified CONSORT[40] flow diagram for individual randomized controlled trial REFYO-R of nonpharmacological treatment
